# Infection Rate and Tissue Localization of Murine IL-12p40-Producing Monocyte-Derived CD103^+^ Lung Dendritic Cells during Pulmonary Tuberculosis

**DOI:** 10.1371/journal.pone.0069287

**Published:** 2013-07-08

**Authors:** Chaniya Leepiyasakulchai, Chato Taher, Olga D. Chuquimia, Jolanta Mazurek, Cecilia Söderberg-Naucler, Carmen Fernández, Markus Sköld

**Affiliations:** 1 Department of Microbiology, Tumor and Cell Biology, Karolinska Institutet, Stockholm, Sweden; 2 Department of Medicine, Solna, Center for Molecular Medicine, Karolinska Institutet, Stockholm, Sweden; 3 Department of Immunology, Wenner-Gren Institute, Stockholm University, Stockholm, Sweden; Fundació Institut d’Investigació en Ciències de la Salut Germans Trias i Pujol. Universitat Autònoma de Barcelona. CIBERES, Spain

## Abstract

Non-hematopoietic cells, including lung epithelial cells, influence host immune responses. By co-culturing primary alveolar epithelial cells and monocytes from naïve donor mice, we show that alveolar epithelial cells support monocyte survival and differentiation in vitro, suggesting a role for non-hematopoietic cells in monocyte differentiation during the steady state in vivo. CD103^+^ dendritic cells (αE-DC) are present at mucosal surfaces. Using a murine primary monocyte adoptive transfer model, we demonstrate that αE-DC in the lungs and pulmonary lymph nodes are monocyte-derived during pulmonary tuberculosis. The tissue localization may influence the functional potential of αE-DC that accumulate in *Mycobacterium tuberculosis*-infected lungs. Here, we confirm the localization of αE-DC in uninfected mice beneath the bronchial epithelial cell layer and near the vascular wall, and show that αE-DC have a similar distribution in the lungs during pulmonary tuberculosis and are detected in the bronchoalveolar lavage fluid from infected mice. Lung DC can be targeted by *M. tuberculosis* in vivo and play a role in bacterial dissemination to the draining lymph node. In contrast to other DC subsets, only a fraction of lung αE-DC are infected with the bacterium. We also show that virulent *M. tuberculosis* does not significantly alter cell surface expression levels of MHC class II on infected cells in vivo and that αE-DC contain the highest frequency of IL-12p40^+^ cells among the myeloid cell subsets in infected lungs. Our results support a model in which inflammatory monocytes are recruited into the *M. tuberculosis*-infected lung tissue and, depending on which non-hematopoietic cells they interact with, differentiate along different paths to give rise to multiple monocyte-derived cells, including DC with a distinctive αE-DC phenotype.

## Introduction

Pulmonary tuberculosis (TB) is a leading cause of death from infectious disease in the world and remains a global threat to public health [1]. Alveolar macrophages (Mϕ) are initial targets for *Mycobacterium tuberculosis*, which later spreads to other myeloid cell subsets in the infected lung tissue, many of which originate from recruited Ly6C^+^ inflammatory monocytes [2-5]. Ly6C^+^ monocyte formation in the bone marrow [6,7], followed by release into circulation and subsequent recruitment and differentiation into various dendritic cell (DC) and Mϕ subsets in mycobacterial-infected tissues is important for anti-bacterial effector functions mediated by innate and adaptive immune cells, and for disease outcome in mice and in humans [3-5,8-14]. Reduced number of monocytes in peripheral blood correlates with increased susceptibility to *M. tuberculosis* and to the live vaccine strain Bacille Calmette–Guérin (BCG) [10,11,13]. A likely explanation for this is the numerous functions monocyte-derived cells have in host immunity in response to mycobacterial infections [3]. Infected monocyte-derived Mϕ have direct bactericidal effector functions mediated by for example inducible nitric oxide synthase (iNOS) [3,8,14]. In addition, DC can be divided into several functionally distinct subsets, including CD103^+^ DC (αE-DC) in the lungs that have a skewed cytokine profile during pulmonary TB [15,16]. αE-DC development depends on the transcription factors IRF8 and Batf3 [17]. In support of an important role for DC in controlling mycobacterial infections, IRF8-deficiency increase susceptibility in humans and in animal models [10,12].

Moreover, DC can activate *M. tuberculosis*-specific T cells in secondary lymphoid organs [9]. Differentiation of IFN-γ-producing *M. tuberculosis*-specific T helper 1 (Th1) cells is dependent on IL-12p40 secreted by DC, and both Th1 cells and IL-12p40 is required for control of bacterial growth [18-21]. In the present study we show that primary alveolar epithelial cells (AEC) from naïve donor mice support monocyte survival and differentiation in vitro, and that recruited monocytes can differentiate into αE-DC in *M. tuberculosis*-infected lungs and in the draining pulmonary lymph node (PLN). Once recruited into infected lungs, αE-DC localize near the bronchial epithelial cell layer and in close proximity to the vascular wall. A small number of αE-DC are also found in the bronchoalveolar lavage (BAL) fluid from *M. tuberculosis*-infected mice. We also confirm that several myeloid cell subsets are targeted by *M. tuberculosis* during the peak of the immune response, and despite localizing in close proximity to the airways only a small fraction of lung αE-DC is infected with *M. tuberculosis* in vivo [2]. As expected, *M. tuberculosis*-infected myeloid cells that localized in the lungs did not downregulate cell surface expression of MHC class II during the first month of infection [2,22]. Finally, we extend our previous findings on the cytokine profile of lung αE-DC during TB and show that αE-DC contain a high percentage of IL-12p40-producing cells suggesting a role for αE-DC in Th1 cell priming [16].

## Materials and Methods

### Ethics Statement

All animal experiments were conducted in accordance with the Swedish Animal Welfare Act. Karolinska Institutet and the Stockholm North Ethical Committee, the Swedish Board of Agriculture approved all animal experiments involving *M. tuberculosis* (permit number N369/10). In some experiments, uninfected animals were housed under pathogen-free conditions at the Animal Department of the Arrhenius Laboratories, Stockholm University, Sweden. The experiments were performed in accordance with the guidelines of the Animal Research Ethics Board at Stockholm University (permit number N27/10).

In all animal experiments, the health status of the mice was monitored daily by animal care technicians or veterinarians to ensure humane treatment.

### Mice

Female C57BL/6 and BALB/c mice (6-9 weeks old) were purchased from Charles River (Germany). C57BL/6 mice expressing the CD45.1 allele of the CD45 molecule were obtained from the animal facility at the Department of Microbiology, Tumor and Cell Biology, Karolinska Institutet.

For experiments involving primary AEC, 8-12-week old female C57BL/6 mice were purchased from NOVA-SCB, Sweden, and TLR4^-/-^ mice were obtained from Karolinska Institutet with the permission of S. Akira (Osaka University, Japan) [23].

### *M. tuberculosis* aerosol infection

The clinical *M. tuberculosis* isolate, strain Harlingen, used for the *M. tuberculosis* aerosol infections was kindly provided by Dr. J. van Embden, National Institute of Public Health and the Environment, The Netherlands [24]. GFP-expressing *M. tuberculosis*, strain H37Rv, was kindly provided by Dr. M. Lerm, Linköping University [25,26]. *M. tuberculosis* aerosol infection were performed as previously described [16]. In brief, frozen aliquots were thawed and bacterial clumps were dispersed. The bacteria were diluted to 1×10^6^ CFU/ml in sterile PBS, 0.02% Tween 80, and placed in a nebulizer (MiniHeart Lo-Flo Nebulizer, Westmed, Tucson, AZ). The animals were infected with a low-dose of *M. tuberculosis* via the respiratory route using a nose-only exposure system (In-Tox Products, Moriarty, NM) calibrated to deliver 20-200 colony-forming units (CFU) into the lungs. The animals used in this study were infected and housed under specific pathogen-free conditions in a biosafety level-3 animal facility at the Astrid Fagraeus Laboratory, Karolinska Institutet.

### CFU determination

The mice were anesthetized by exposure to isoflurane and euthanized by cervical dislocation. Both lungs were used for day one CFU determinations. Viable mycobacteria were quantified by plating the lung homogenates onto Middlebrook 7H11 agar plates. Colonies were counted after 2-3 weeks of incubation at 37°C.

### Monocyte adoptive transfer into *M. tuberculosis*-infected recipient mice

1×10^6^ primary monocytes were enriched from bone marrow of naïve donor C57BL/6 mice and adoptively transferred into *M. tuberculosis*-infected (Harlingen strain) C57BL/6.CD45.1 recipient mice as previously described [3]. Adoptive transfers were performed three weeks post infection (p.i.). Single cell suspensions were prepared from total lung tissue and PLN at day 10 after cell transfer. The cell surface phenotype of CD45.2^+^ transferred cells was analyzed using flow cytometry as described below. All recipient mice were analyzed individually.

### Immunohistochemistry

Lung tissue from naïve or *M. tuberculosis*-infected mice were perfused with PBS and cut into smaller pieces. The tissue samples were fixed in 4% paraformaldehyde for 2h and dehydrated in 30% sucrose at 4°C overnight before embedding in OCT freezing media (Thermo Scientific). 5-µm sections were cut using a cryostat (Microm HM550, Thermo Scientific) and adhered to Superfrost Plus slides (Thermo Scientific). Sections were kept at -20°C until use.

The sections were dried for 20 minutes at room temperature (RT) before blocking with 2% normal mouse serum in common Ab diluent (BioGenex) and Tris buffer containing 0.01% Triton X-100 (Sigma Aldrich) for 30 minutes at RT before washing and subsequent blocking using Dako protein block (Dako) for 30 minutes at RT. An unconjugated primary rat anti-mouse MHC class II (M5/114.15.2) mAb, or an isotype control mAb (BD bioscience), was added and incubated for 1h at RT. 2% goat serum was then used for blocking (30 minutes at RT), followed by staining with a secondary goat anti-rat IgG (H+L)-Alexa Fluor 633 mAb (Invitrogen) for 1 hour at RT. Next, the sections where then blocked with 2% normal rat serum prior to staining with anti-mouse CD103-PE (M290), or an isotype control mAb (BD Biosciences) for 2h at RT. Nuclei were detected with 4',6-diamidino-2-phenylindole (DAPI) (Invitrogen). Stained slides were mounted with fluorescence mounting medium (Dako) and images were acquired using a Leica TCS SP5 II confocal microscope (Leica).

### Preparation of single-cell suspensions

At the indicated timepoints, single-cell suspensions were prepared from lungs and PLN. The mice were euthanized and blood removed from the lung tissue by perfusing the heart with PBS. Lungs and PLN were aseptically removed and placed in RPMI 1640 medium. The lungs were cut into small pieces and incubated in complete RPMI 1640 medium (supplemented with 10% fetal calf serum, penicillin/streptomycin, L-glutamine, sodium-pyruvate and HEPES buffer, all from Sigma-Aldrich) containing 140 U/ml collagenase type IV (Sigma-Aldrich) for 90 minutes at 37^°^C, 5% CO_2_. DNase I (Sigma-Aldrich) was added to the cell suspensions, at a final concentration of 200 U/ml, during the last 10 minutes of the incubation. The digested lung tissue was then passed through steel mesh cup sieve (Sigma-Aldrich). Any remaining erythrocytes were lysed using a lysis buffer (H_2_O, 0.15 M NH_4_Cl, 1 mM KHCO_3_, 0.1 mM NaEDTA, pH 7.2-7.4), washed and resuspended in RPMI 1640 medium. The cell suspension was passed through a 70-µm cell strainer (BD Falcon), washed and resuspended in complete RPMI 1640 medium.

BAL was collected from euthanized BALB/c mice by delivering 1.5 ml of PBS through the tracheal tube using a 18 gauge needle. The fluid was gently drawn back immediately after delivery. The cells in 0.75-1.2 ml of BAL were collected by centrifugation, counted and analyzed by flow cytometry.

Single-cell suspensions were obtained from PLN using collagenase type IV and DNAse I as described above. The PLN were then disaggregated using the frosted ends of two glass slides, washed and resuspended in complete RPMI 1640 medium.

Total viable cells were enumerated using a hemocytometer and trypan blue exclusion of dead cells.

### Primary AEC and monocyte in vitro co-culture

Primary AEC, containing both type I and type II AEC, were enriched from naive donor mice as described by Chuquimia et al [27]. First, the lungs were perfused with RPMI medium to remove red blood cells, followed by installation of dispase (Gibco-Invitrogen) into the lungs via the trachea. The lungs were then removed and digested using dispase for 45 minutes at RT. The single-cell suspension that was obtained by loosening the parenchymal tissue was treated with DNAse I (Sigma) for 30 minutes at RT, and passed through 70 µm and 40 µm cell strainers. Remaining RBC were lysed as described above. To obtain AEC, the cell suspension was then depleted of CD45^+^ and CD146^+^ cells using magnetic cell sorting with LD separation columns (Miltenyi Biotec). Enriched AEC (5×10^4^ cells/well) were cultured in complete RPMI 1640 medium at 37^°^C, 5% CO_2_ for two days. Non-adherent cells were removed before primary bone marrow monocytes were added in complete RPMI 1640 medium at a monocyte/AEC-ratio of 5:1. In some experiments, primary WT or TLR4^-/-^ monocytes were cultured with AEC-derived media (1/2 final dilution) obtained from untreated, or LPS-stimulated AEC as described previously [28]. Monocyte differentiation was examined after 3-10 days as indicated.

A commercially available ELISA kit (R&D Systems) was used according to the manufacturer’s instructions to determine the GM-CSF-levels in unstimulated AEC culture supernatants.

### Flow cytometry

Staining for surface markers was done by resuspending 2×10^6^ cells in FACS buffer (PBS with 1% (w/v) BSA and 2 mM NaN_3_). The cells were incubated with purified anti-mouse CD16/CD32 (2.4G2, BD Pharmingen) at 10 µg/ml for 15 min at 4°C to block nonspecific binding. The cells were washed and incubated for 15 minutes at 4°C with primary mAbs, or appropriate isotype control mAbs, diluted in FACS buffer. The anti-CD103 (M290)-PE and anti-CD45.1 (A20)-FITC mAbs were purchased from BD Biosciences. Streptavidin-Pacific Orange was purchased from Invitrogen. The following PE-, PE-Cy5.5-, PE-Cy7, APC-, AlexaFluor 700-conjugated or biotinylated anti-mouse mAbs were purchased from eBioscience: anti-CD45.2 (104), anti-CD11c (N418), anti-CD11b (M1/70), anti-CD19 (1D3). Anti-I-A/I-E-PerCP (M5/114.15.2) was purchased from Biolegend. Stained cells were washed and fixed in freshly prepared 2% paraformaldehyde in PBS for 2h at 4°C. Fixed cells were washed and resuspended in FACS buffer before analysis.

For detection of iNOS-producing cells, the fixed cells were permeabilized for 30 minutes at RT using a Cytofix/Cytoperm™ kit from BD Biosciences. Intracellular iNOS was detected using an unconjugated anti-iNOS-mAb (clone 6, BD Transduction Laboratories) coupled to the Zenon complex-Alexa Fluoro 647 (Invitrogen) according to the manufacturer’s instructions, and incubated for 30 minutes at RT. Specific iNOS-staining was compared to a relevant isotype control mAb (BD Transduction Laboratories). Stained cells were washed and analyzed immediately by flow cytometry.

The cells were collected using a BD LSR II or a BD FACSCanto (BD Biosciences) and analyzed using FlowJo software (version 8.8.6, Tree Star). All gates and quadrants were set after relevant isotype control mAbs.

### In vitro stimulation of lung cells and intracellular cytokine staining

Single cell suspensions were prepared from *M. tuberculosis*-infected mice and kept in complete RPMI 1640 medium, or stimulated with 100 ng/ml *E. coli* LPS (Sigma-Aldrich) or 10 µg/ml *M. tuberculosis* cell wall extract (prepared as previously described [16]) in the presence of 10 µg/ml Brefeldin A (Sigma-Aldrich) for 5h at 37^°^C, 5% CO_2_.

Adherent cells were detached by incubating the cells in PBS, 2 mM EDTA, for 10 minutes at 37^°^C, 5% CO_2_. The cells were stained for the indicated cell surface markers, fixed in 2% paraformaldehyde, permeabilized and stained for the intracellular cytokines IL-10-FITC (JES5-16E3, eBioscience) and IL-12-APC (C15.6, BD Bioscience) or relevant isotype control mAbs. Stained cells were washed twice in permeabilization buffer and once with FACS buffer and analyzed immediately.

## Results

### Primary AEC support monocyte survival and differentiation in vitro

**Figure 1:**
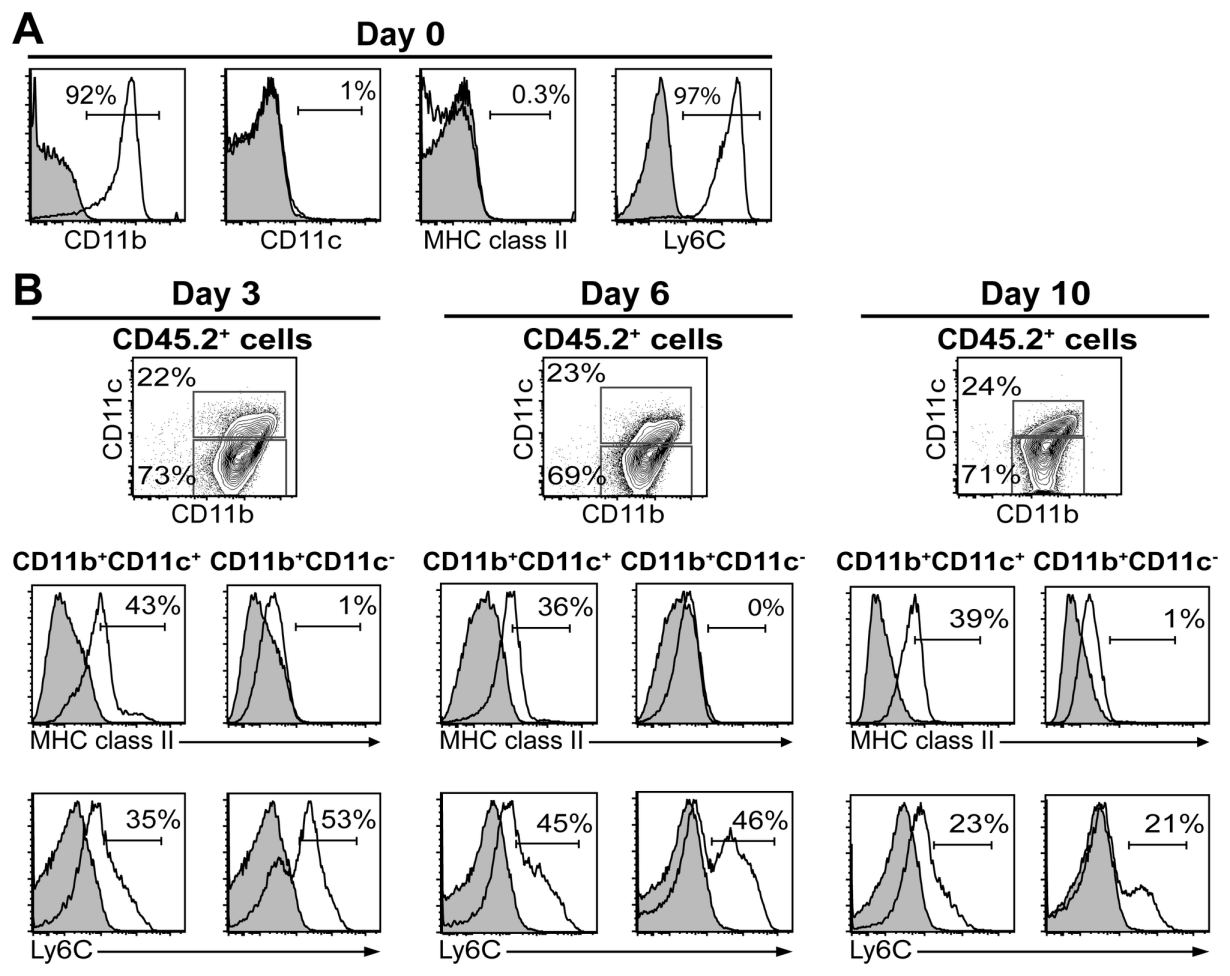
Primary monocytes survive and differentiate when cultured with AEC in vitro. Primary monocytes and AEC were enriched from naïve donor mice and co-cultured for three, six or ten days. (A) The histograms show the phenotype of the enriched monocytes used in the co-culture experiments. (B) The cells were harvested at the indicated timepoints and stained for cell surface markers. Cultured myeloid cells were identified based on CD45.2 cell surface expression (data not shown) and analyzed for CD11b and CD11c expression (upper panels). The lower panels display MHC class II and Ly6C cell surface expression (open histograms) on gated CD11b^+^CD11c^+^ and CD11b^+^CD11c^-^ monocyte-derived cells. Filled histograms show isotype control stainings. One representative experiment out of three separate experiments is shown.

Because myeloid cells reside in close proximity to AEC we investigated if AEC, or AEC-derived soluble factors, support monocyte differentiation in vitro. Primary monocytes and AEC were purified as previously described and co-cultured in vitro for three, six or ten days as outlined in Materials and Methods (figure 1) [3,27]. Alternatively, primary WT or TLR4^-/-^ monocytes were cultured alone in AEC-conditioned media from untreated AEC, or from LPS-stimulated AEC, respectively (data not shown). The supernatant from untreated AEC contain detectable amounts of several cytokines and chemokines, for example GM-CSF and MCP-1 [27]. After 24 h, we detected 464 pg/ml of GM-CSF in the supernatants from unstimulated AEC used in this study. The range and the amounts of the various soluble factors produced by AEC is markedly increased after LPS stimulation. For example, LPS increases GM-CSF production twofold [27]. In contrast, the M-CSF levels were undetectable by ELISA in the supernatants from unstimulated and from LPS-stimulated AEC [27].

Ly6C^+^ bone marrow monocytes have the same cell surface phenotype as inflammatory monocytes in circulation and were used in the in vitro co-culture experiments, and in the adoptive transfer experiments described below, as a substitute for Ly6C^+^ inflammatory monocytes in peripheral blood [3,5]. Primary monocytes enriched from the bone marrow also express CD11b, but lack expression of CD11c, MHC class II and CD103 (Figure 1A and data not shown) [3]. Monocytes cultured in media alone did not survive, and preliminary data suggests that monocytes cultured in media supplemented with either AEC supernatant did not change CD11c, MHC class II or CD103 cell surface expression (data not shown). In contrast, monocytes cultured in contact with primary AEC upregulated low levels of CD11c within three days (Figure 1B). The percentage of CD11c^+^ cells and the CD11c surface expression levels remained constant during the whole experiment. We also investigated CD11b^+^CD11c^+^ and CD11b^+^CD11c^-^ monocyte-derived cells for MHC class II and Ly6C cell surface expression (Figure 1B). While CD11b^+^CD11c^-^ cells remained MHC class II^-^, CD11b^+^CD11c^+^ cells expressed low levels of MHC class II. We also observed that CD11b^+^CD11c^+^ cells expressed lower levels of Ly6C than CD11b^+^CD11c^-^ cells. Even though the latter population did not upregulate CD11c or MHC class II, the fraction of Ly6C-expressing cells decreased from around 50% to approximately 20% by day 10, suggesting a more differentiated phenotype. Finally, we did not detect CD103 expression by any myeloid cell subset in the in vitro co-cultures (data not shown).

To the best of our knowledge it has not previously been shown that primary AEC interaction with primary monocytes in vitro support monocyte survival and differentiation without the addition of exogenous factors.

### Recruited monocytes differentiate into lung αE-DC during pulmonary TB

Several lung Mϕ and DC subsets originate from monocytes recruited into the inflamed lung tissue from peripheral blood [3]. To determine whether recruited monocytes are precursor cells to lung αE-DC during pulmonary TB and contribute to the increase in cell numbers in response to infection we took advantage of a monocyte adoptive transfer model using enriched primary bone marrow monocytes from naïve donor mice [3,16].

**Figure 2:**
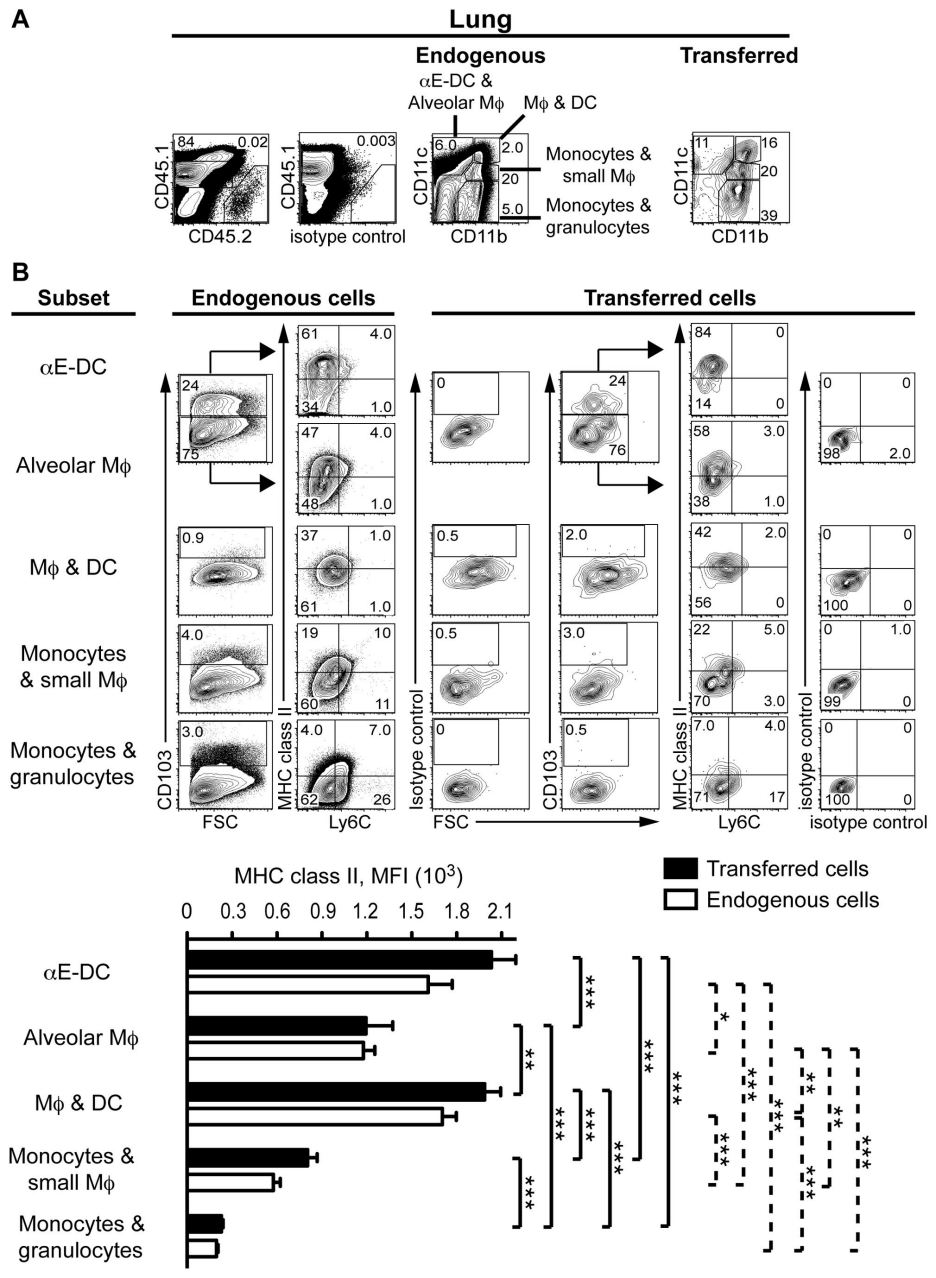
Recruited inflammatory monocytes differentiate into lung αE-DC during pulmonary TB. Monocytes enriched from naïve CD45.2^+^ C57BL/6 mice were adoptively transferred into *M. tuberculosis*-infected CD45.1^+^ congenic recipient mice (three weeks p.i.). Endogenous and transferred cells in lung tissue were identified and analyzed 10 days later. (A) Donor cells were identified based on CD45.2 expression compared to an isotype control mAb (left panels). Several myeloid cell subsets were then identified in gated CD45.1^+^ endogenous and CD45.2^+^ transferred cells based on the CD11b and CD11c expression profile (right panels). Endogenous αE-DC and alveolar Mϕ were identified among CD11b^-^CD11c^+^ cells, while CD11b^+^CD11c^+^ cells contain Mϕ and DC. Endogenous CD11b ^intermediate^CD11c^intermediate^ cells are monocytes and small Mϕ, and endogenous CD11b^+^CD11c^-^ cells contain monocytes and granulocytes. The corresponding myeloid subsets were identified among transferred cells. (B) Each myeloid subset was analyzed for CD103, Ly6C and MHC class II cell surface expression. CD103-expressing Ly6C^-^MHC class II^+^ αE-DC were identified in the CD11b^-^CD11c^+^ gate among both endogenous and transferred cells (upper panels). The graph displays MHC class II cell surface expression levels (mean ± SEM) on gated transferred and endogenous cells. *, p<0.05; **, p<0.01; ***, p<0.001 by one-way ANOVA with Bonferroni posttest. Solid lines denote comparisons between transferred cells and dotted lines denote comparisons between endogenous cells. Monocyte differentiation in *M. tuberculosis*-infected recipient mice was analyzed in two separate experiments with five recipient mice in each experiment.

We have previously observed that a monocyte-derived CD11b^-^CD11c^+^ population expressing high levels of MHC class II start to appear in *M. tuberculosis* infected lungs around day six after monocyte adoptive transfer. This myeloid cell subset lacked cell surface expression of typical Mϕ markers [3]. We therefore waited 10 days before we analyzed the recipient mice for monocyte-derived cells (Figure 2). Recipient cells were identified based on CD45.1 cell surface expression. The CD45.1^high^ lung cells are mostly autofluorescent alveolar Mϕ and were excluded in the analysis to enable easy identification of αE-DC. Donor cells were identified based on CD45.2 cell surface expression compared to an isotype control mAb (Figure 2A). The CD11b/CD11c cell surface expression profile of endogenous and monocyte-derived cells enabled us to identify several myeloid cell subsets [3,29]. The various subsets were then analyzed further based on Ly6C, MHC class II and CD103 expression. The CD11b^+^CD11c^-^ subset contains a significant proportion of Ly6C^+^ cells expressing low levels of MHC class II and few CD103^+^ cells, and is a mixture of monocytes and small Mϕ [3,29]. In addition, the endogenous CD11b^+^CD11c^-^ cell population also contained granulocytes [3,29]. Recruited monocytes rapidly upregulate CD11c in *M. tuberculosis*-infected lung tissue [3]. The CD11b^+^CD11c^intermediate^ population contains few Ly6C^+^ cells, but a higher percentage of MHC class II^+^ cells compared to the CD11b^+^CD11c^-^ subset. CD11b^+^CD11c^intermediate^ cells do not express CD103 and are monocytes and small Mϕ [3,29]. In contrast to monocytes differentiating in uninfected lung tissue, monocytes in *M. tuberculosis*-infected lungs form a large fraction of CD11b^+^CD11c^+^ cells. This subset has downregulated Ly6C, contain a high percentage of MHC class II^+^ cells, but no CD103^+^ cells. The CD11b^+^CD11c^+^ subset contain both activated iNOS^+^ Mϕ, and DC [3,29]. Finally, endogenous CD11b^-^CD11c^+^ cells contain a fraction of autofluorescent alveolar Mϕ and CD103^+^ αE-DC [15,29]. Both subsets lack expression of Ly6C, but αE-DC express higher cell surface levels of MHC class II. Recruited monocytes also give rise to CD11b^-^CD11c^+^ cells that are Ly6C^-^, CD103^+^ and express high levels of MHC class II, i.e. αE-DC (Figure 2). It is noteworthy that CD11b^-^CD11c^+^ cells are the only myeloid subset containing a significant proportion of CD103^+^ cells. At present, it is not known if the monocyte-derived CD11b^-^CD11c^+^CD103^-^ population contains alveolar Mϕ in the *M. tuberculosis*-infected lungs.

Our results show for the first time that recruited inflammatory monocytes can differentiate into CD11b^-^CD11c^+^Ly6C^-^CD103^+^MHC class II^+^ αE-DC in the lung tissue during pulmonary TB.

### Recruited monocytes give rise to αE-DC in lymph nodes draining the lung tissue during TB

**Figure 3:**
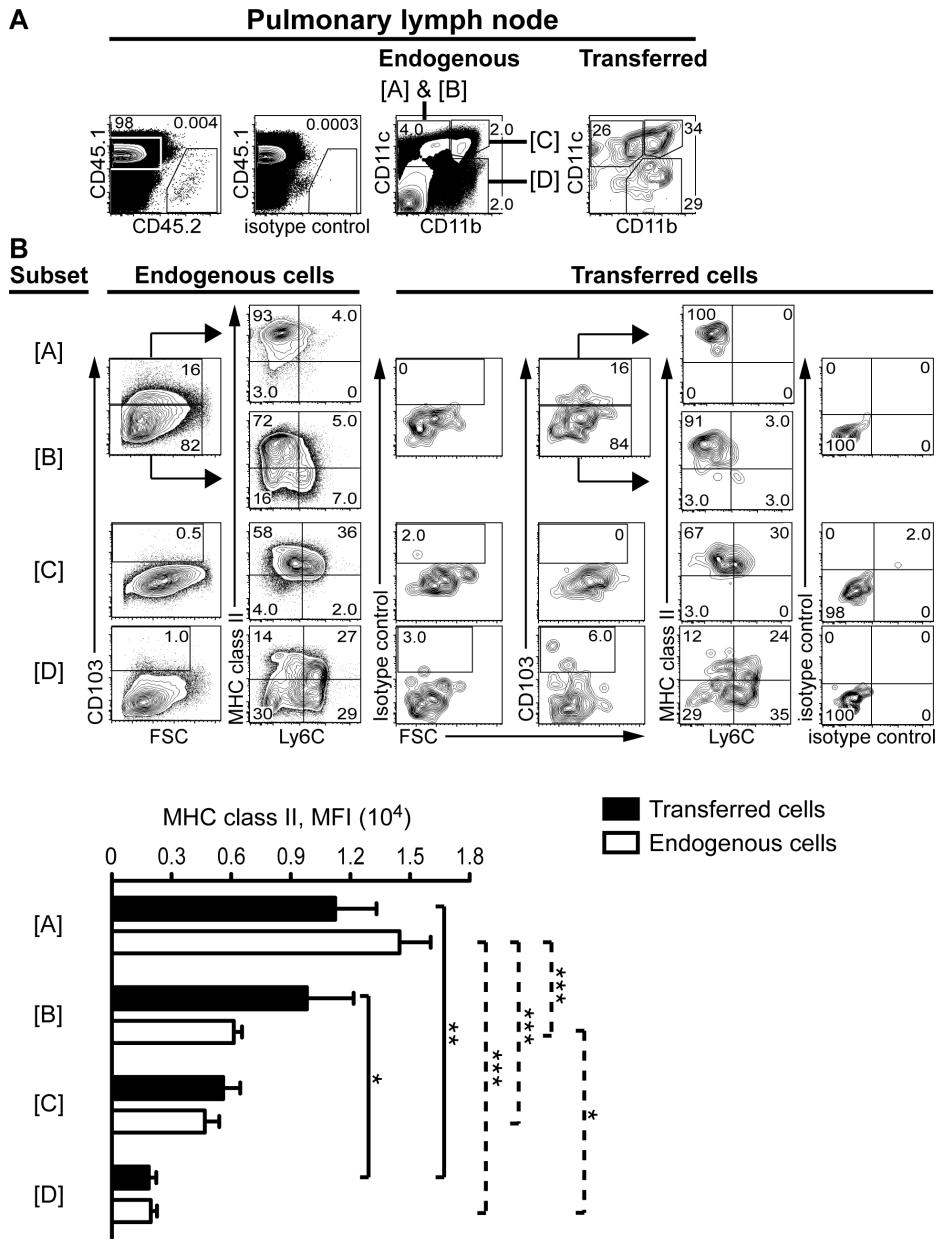
Recruited inflammatory monocytes give rise to αE-DC in the draining PLN during TB. (A) Primary monocytes were adoptively transferred and identified in infected recipient mice as described in Figure 1 (upper left panels). The myeloid cell subsets were identified based on CD11b and CD11c expression profile (labeled [A]-[D], upper right panels). (B) The myeloid cell subsets were analyzed further for CD103, Ly6C and MHC class II cell surface expression. CD103-expressing Ly6C^-^MHC class II^+^ αE-DC were found within the CD11b^-^CD11c^+^ population (upper panels). The graph shows MHC class II cell surface expression levels (mean ± SEM) on gated transferred and endogenous cells. *, p<0.05; **, p<0.01; ***, p<0.001 by one-way ANOVA with Bonferroni posttest. Solid lines denote comparisons between transferred cells and dotted lines denote comparisons between endogenous cells. Monocyte differentiation in *M. tuberculosis*-infected recipient mice was analyzed in two separate experiments with five recipient mice in each experiment.

Similar to the lungs, inflammatory monocytes contribute to several myeloid cell subsets in the PLN following *M. tuberculosis* infection [3]. We used our monocyte adoptive transfer model to determine if αE-DC in the PLN are monocyte-derived during pulmonary TB (Figure 3). Similar to the lung tissue, the CD11b/CD11c expression profile was used to divide the myeloid cell compartment into three subsets (Figure 3A). We then compared CD45.1^+^ endogenous cells and CD45.2^+^ transferred cells in infected recipient mice. The only endogenous myeloid cell subset that contained a significant proportion of CD103^+^ cells did not express CD11b but was CD11c^+^. The CD11b^-^CD11c^+^CD103^+^ cells were Ly6C^-^ and MHC class II^high^ (Figure 3B).

Approximately one third of the recruited monocytes were CD11b^+^CD11c^-^ on day 10 after adoptive transfer (Figure 3A). This subset was CD103^-^, most cells expressed Ly6C^+^ and low cell surface levels of MHC class II compared to isotype control mAbs (Figure 3B). One third of the monocyte-derived cells were CD11b^+^CD11c^+^ that did not express CD103, expressed low levels of Ly6C and were MHC class II^+^ (Figure 3). This subset contains myeloid DC and MAC-3^+^ iNOS-producing cells [2,3]. Finally, one third of the monocyte-derived cells expressed CD11c, but not CD11b (Figure 3A). CD103^+^ cells were identified in this CD11b^-^CD11c^+^ subset that were Ly6C^-^ and expressed high cell surface levels of MHC class II (Figure 3B).

Our original finding shows that inflammatory monocytes can give rise to αE-DC that appear in the PLN draining the lung tissue during pulmonary TB.

### αE-DC localize beneath the bronchial epithelial cell layer and near the vascular wall in *M. tuberculosis*-infected lungs

**Figure 4:**
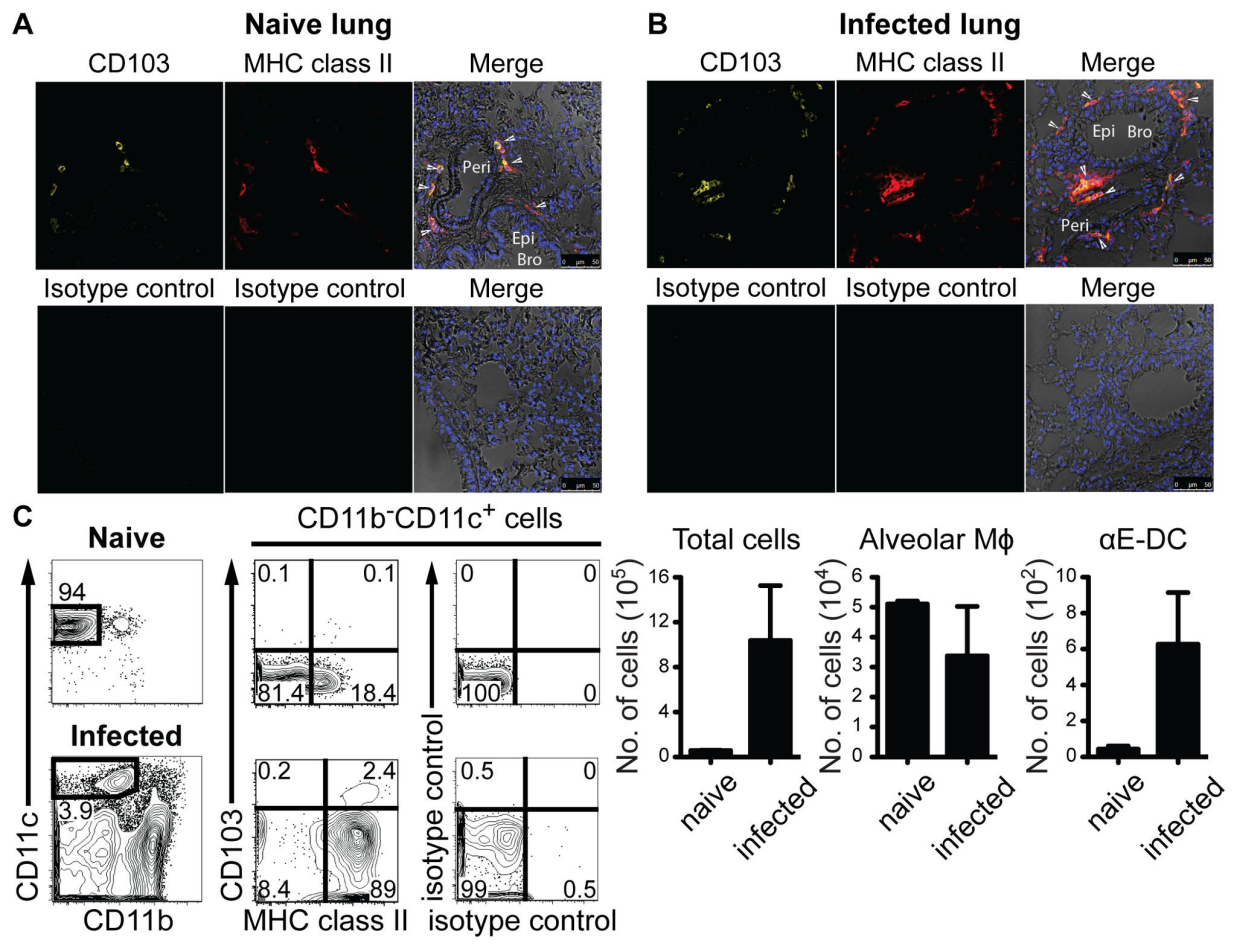
Tissue localization of lung αE-DC in uninfected mice and during pulmonary TB. Fixed lung tissue sections from C57BL/6 mice were stained with DAPI, and with CD103 and MHC class II mAbs, or isotype controls, to identify DAPI ^+^ CD103^+^MHC class II^+^ αE-DC in uninfected (A) or *M. tuberculosis*-infected (three weeks p.i.) (B) lungs. Arrowheads indicate the localization of some lung αE-DC. αE-DC localization in the lungs was determined in five separate experiments. Peri = Perivascular wall, Epi = Epithelium, Bro = Bronchus. (C) The contour-plots show identification of CD11b^-^CD11c^+^ cells (pre-gated on CD45.2 ^+^ CD19^-^ cells, data not shown) in BAL collected from 10 pooled uninfected (upper panels) BALB/c mice, or a *M. tuberculosis*-infected (18 weeks p.i., lower panels) BALB/c mouse. Gated CD11b^-^CD11c^+^ cells were analyzed further for CD103 and MHC class II cell surface expression. The graphs display the absolute number of cells (mean ± SD) in BAL fluid from uninfected and infected animals. Alveolar Mϕ and αE-DC in BAL were examined in two separate experiments.

Sung et al first identified murine αE-DC in the lung tissue, and showed that αE-DC localize near the basal surface of bronchial epithelial cells and in close proximity to vascular endothelial cells [15]. To confirm the tissue localization of αE-DC in naïve mice, we used the co-expression pattern of CD103 and MHC class II as a criterion to distinguish αE-DC from other lymphoid and myeloid cell subsets in lung tissue sections (Figure 4). As expected CD103^+^MHC class II^+^ αE-DC localize in close proximity to the basolateral side of the bronchial epithelial cell layer in naïve mouse lungs. Also, CD103^+^MHC class II^+^ αE-DC were identified close to the arterial wall (Figure 4A).

We have shown that the functional potential of αE-DC was preserved during early and chronic *M. tuberculosis* infection [16]. Since the localization near the bronchial epithelium may contribute to αE-DC function during TB, we determined if αE-DC tissue localization change in response to *M. tuberculosis* infection. Figure 4B identifies CD103^+^ MHC class II^+^ αE-DC in *M. tuberculosis*-infected lungs three weeks p.i. At this timepoint, the absolute number of lung αE-DC increases almost 15-fold in resistant C57BL/6 and BALB/c mice in response to *M. tuberculosis* infection compared to naïve animals [16]. Similar to naïve mice, αE-DC localize near the basolateral side of the bronchial epithelium and close to the arterial vasculature in *M. tuberculosis*-infected mice.

αE-DC localization near the airways during TB prompted us to investigate if αE-DC were detected in BAL fluid collected from naïve or *M. tuberculosis*-infected BALB/c mice (Figure 4C). Most cells in the BAL from naïve mice were CD11b^-^CD11c^+^CD103^-^ alveolar Mϕ while αE-DC were essentially undetectable. While the absolute number of total BAL cells increased 17-fold by week 18 p.i., the absolute number of alveolar Mϕ remained unchanged. We also observed that most alveolar Mϕ upregulated MHC class II during TB. In response to *M. tuberculosis* infection αE-DC were detectable in the BAL, but remained a minor cell subset in the BAL fluid (Figure 4C).

In conclusion, while *M. tuberculosis* infection increases the absolute number of lung αE-DC, it does not influence αE-DC tissue localization in the infected lungs with the exception of the BAL fluid, in which the absolute number of αE-DC increased in response to infection The constant localization next to the bronchial epithelium in naïve and infected mice may help explain the conserved functional potential of αE-DC during TB.

### A minor fraction of αE-DC is infected with *M. tuberculosis* in vivo

**Figure 5:**
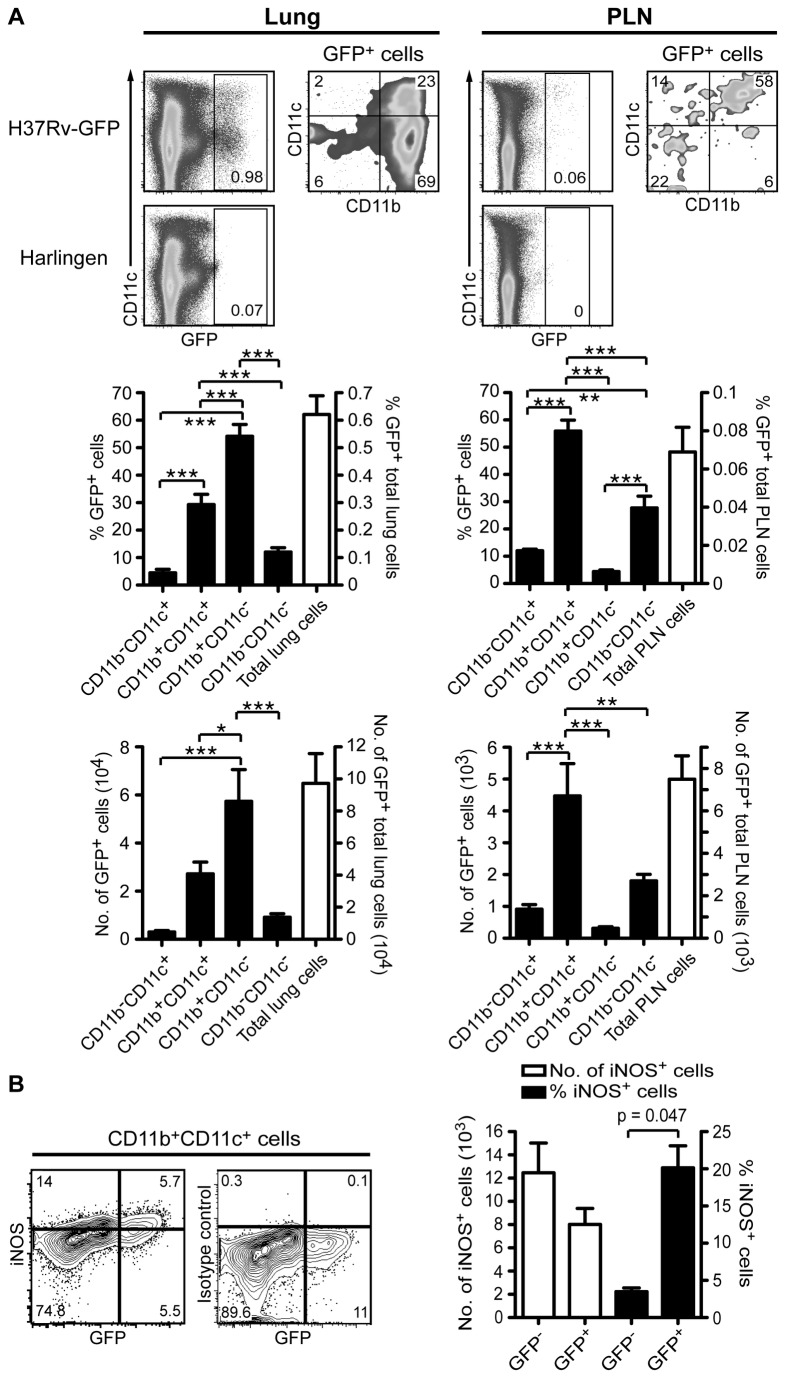
Infected myeloid cells during pulmonary TB. C57BL/6 mice were infected with *M. tuberculosis* (strain H37Rv-GFP, or strain Harlingen) via the respiratory route. Three weeks p.i., total lung and PLN cell suspensions were stained for cell surface markers and infected cells were identified based on GFP expression (pre-gated on CD45.2 ^+^ CD19^-^ cells, data not shown). (A) The contour plots show identification of H37Rv-GFP-infected cells in the lungs (left panels) and in the PLN (right panels). For comparison, the lower plots display lung- and PLN cells infected with *M. tuberculosis* strain Harlingen. The bar graphs display the percentage and absolute number of infected total cells and defined cell subsets in the lungs (left panels) and in the PLN (right panels). The graphs display mean ± SEM. *, p<0.05; **, p<0.01; ***, p<0.001 by one-way ANOVA with Bonferroni posttest. (B) The contour plot shows iNOS- and GFP-expression in gated CD11b^+^CD11c^+^ lung cells (left panel). The iNOS staining was compared to an isotype control mAb (right panel) and GFP-expression was compared to Harlingen-infected lung cells (data not shown). The bar graph displays the absolute number and the percentage of iNOS^+^ CD11b^+^CD11c^+^ cells (mean ± SEM). iNOS-expression is compared in uninfected (GFP^-^) and infected (GFP^+^) cells. A *t* test was used to determine statistical difference between uninfected and infected cells. Infected cells were analyzed in four separate experiments.

**Figure 6:**
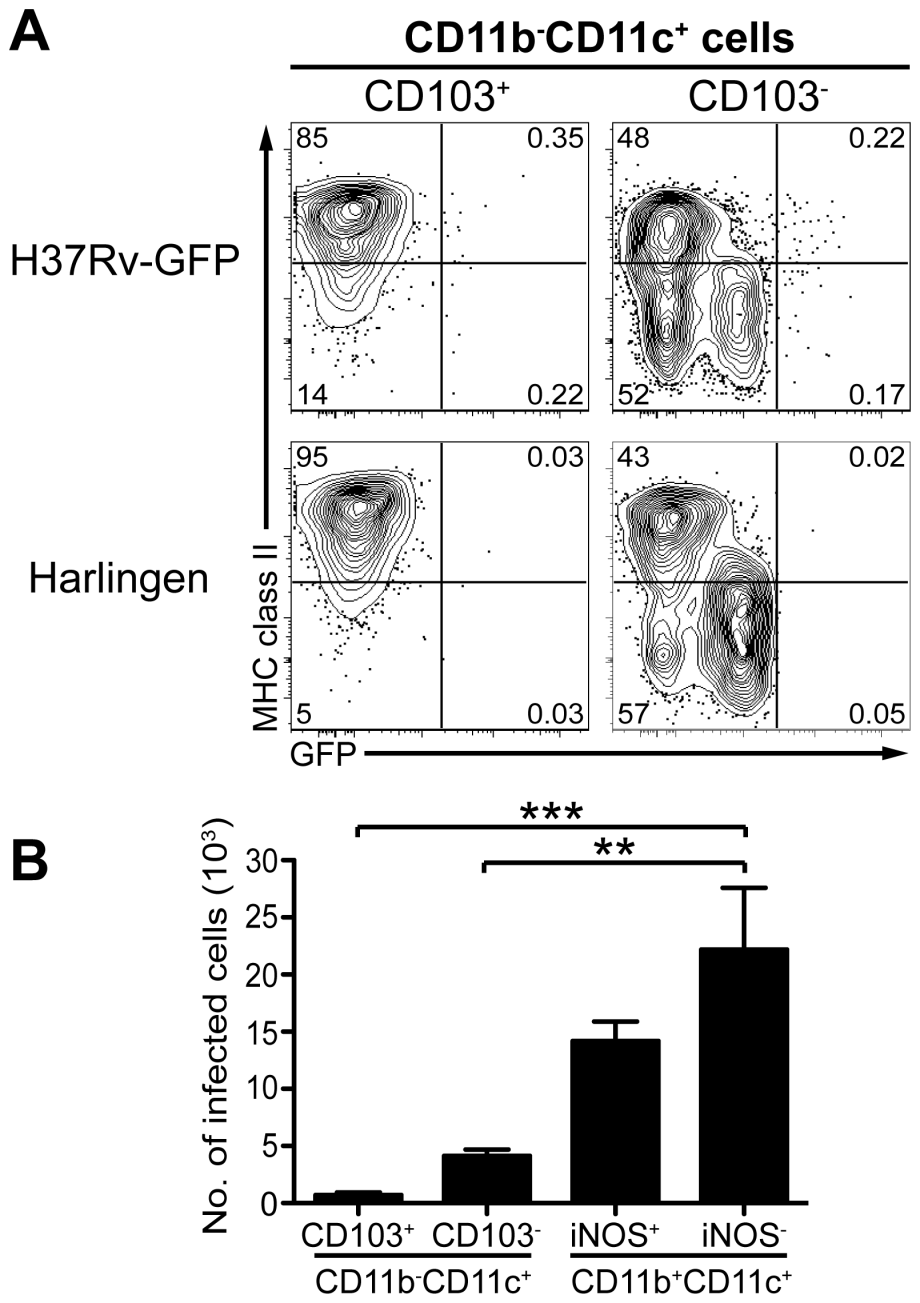
Few *M. tuberculosis*-infected lung αE-DC in vivo. C57BL/6 mice were infected with *M. tuberculosis* strain H37Rv-GFP, or strain Harlingen. αE-DC and alveolar Mϕ were identified in infected lungs three weeks p.i. in the CD11b^-^CD11c^+^ gate as described in Figure 2. (A) The contour plots show GFP- and MHC class II expression by lung αE-DC and alveolar Mϕ from mice infected with H37Rv-GFP (upper panels) or Harlingen (lower panels). The quadrants were set after a relevant isotype control mAb (not shown) and after the GFP^-^ mycobacteria. (B) The graph shows the absolute number of infected αE-DC and alveolar Mϕ three weeks p.i., as well as the absolute number of infected cells among iNOS^-^ and iNOS^+^ CD11b^+^CD11c^-^ cells. The graphs display mean ± SEM. **, p<0.01; ***, p<0.001 by one-way ANOVA with Bonferroni posttest. *M. tuberculosis* infection of lung αE-DC was determined in four independent experiments.

**Figure 7:**
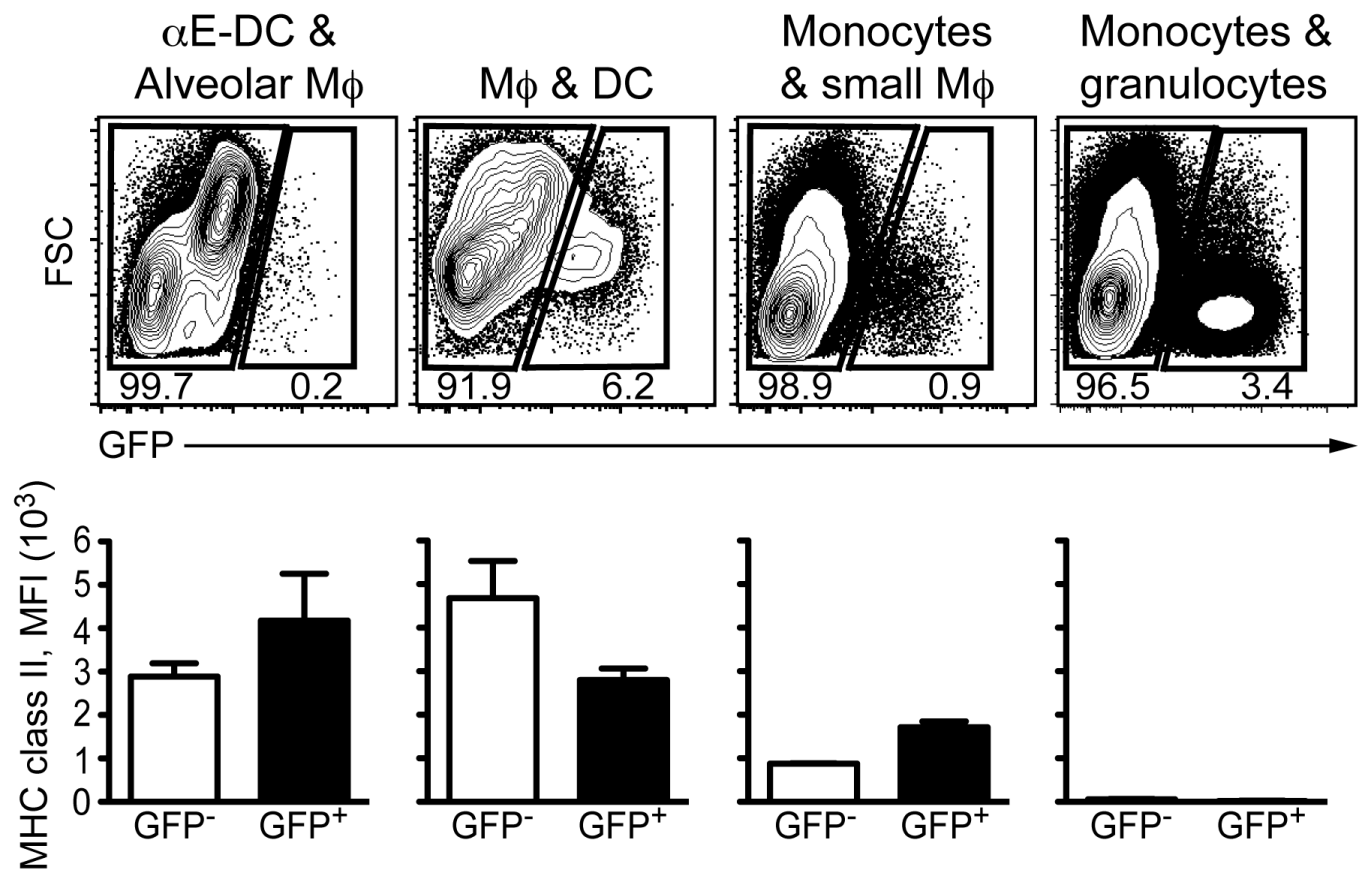
MHC class II expression levels on *M. tuberculosis*-infected myeloid cells. C57BL/6 mice were infected with *M. tuberculosis* H37Rv-GFP via the respiratory route and the indicated myeloid cell subsets were identified in infected lungs as described in Figure 1. Based on GFP expression, uninfected (GFP^-^) and infected (GFP^+^) cells were further analyzed for MHC class II cell surface expression levels. A *t* test was used to determine statistical difference between uninfected and infected cells. One representative experiment out of four separate experiments is shown.

Multiple myeloid cell subsets are infected with *M. tuberculosis* following aerosol infection [2]. Since DC have been implicated in mycobacterial dissemination from the lungs to the draining PLN, we investigated if αE-DC are targeted by *M. tuberculosis* in vivo [2,30]. Three weeks p.i at the peak of the immune response, CD11b^+^CD11c^+^ cells have been shown to be the main target of *M. tuberculosis* in the infected lungs [2]. We used GFP-expressing virulent *M. tuberculosis* strain H37Rv to identify infected myeloid cells, and cells isolated from animals infected with the GFP^-^ *M. tuberculosis* strain Harlingen as a negative control for the gating strategy (figures 5-7). We confirmed that a large fraction of the CD11b^+^CD11c^+^ lung cells are *M. tuberculosis*-infected (Figure 5A). However, the CD11b^+^CD11c^-^ population contained the highest frequency of infected cells. This population of infected cells was MHC class II negative (see Figure 7), but with the current cell surface staining strategy we cannot determine if the cells are infected monocytes or neutrophils [2,3]. Finally, around two percent of infected myeloid lung cells were CD11b^-^CD11c^+^, which include both alveolar Mϕ and αE-DC (Figure 5A).

In agreement with previous observations made three weeks p.i., we found that CD11b^+^CD11c^+^ myeloid DC contain the highest frequency of *M. tuberculosis*-infected cells in the draining PLN (Figure 5A) [2]. We also observed that 12.1 ± 1.7%, n = 9 [mean ± SD] of CD11b^-^CD11c^+^ cells are infected in the PLN. Compared to the lung tissue, the frequency and absolute number of infected cells was approximately 10-fold lower in the PLN (Figure 5A).

Based on morphology, cell surface phenotype and functional potential, CD11b^+^CD11c^+^ cells in *M. tuberculosis*-infected lungs contain both DC and activated Mϕ [3,29]. Also, infected lung cells have been shown to express iNOS, even though the majority of infected cells remained iNOS negative [31]. Using GFP-expressing *M. tuberculosis*, we were able to further delineate the functional potential of infected CD11b^+^CD11c^+^ lung cells (Figure 5B). On average, 20.1% of infected CD11b^+^CD11c^+^ lung cells co-expressed iNOS, supporting the idea that this myeloid cell subset contain infected and activated Mϕ [3]. Our results also show that most iNOS-producing CD11b^+^CD11c^+^ lung cells are not infected with *M. tuberculosis* (Figure 5B).

In figure 6 we investigate if αE-DC are infected with *M. tuberculosis* at week three p.i. Only 0.15 ± 0.088%, n = 16 [mean ± SD] appeared to be infected. As in Figure 5, we compared mice infected with GFP-expressing H37Rv with the GFP-negative strain Harlingen. In comparison 0.54 ± 0.30%, n = 16 [mean ± SD] of CD103^-^CD11b^-^CD11c^+^ cells that contain alveolar Mϕ were infected. Enumeration of infected αE-DC confirms that this DC population is not a major target for the bacterium at this stage of the disease. For comparison, we included the total number of infected iNOS^-^ and iNOS^+^ CD11b^+^CD11c^+^ myeloid cells in the bar graph in Figure 6B. Both CD11b^+^CD11c^+^ cell subsets are to a larger extent than αE-DC infected with *M. tuberculosis* three weeks p.i.

Our results clearly show that virulent *M. tuberculosis* preferentially infects certain myeloid cell subsets that localize in the lungs or in the PLN, while other myeloid cells, including αE-DC, remain essentially uninfected despite the localization near the airways.

### *M. tuberculosis*-infected myeloid cells in the lungs do not down-regulate MHC class II cell surface expression

We took advantage of the GFP-expressing *M. tuberculosis* to determine if infected myeloid cells in the lungs expressed lower cell surface levels of MHC class II. Using the same gating strategy as in Figure 2, we identified myeloid cell subsets containing αE-DC/alveolar Mϕ, DC/Mϕ, monocytes/small Mϕ, and monocytes/granulocytes (Figure 7). Within each subset we distinguished between GFP^-^ uninfected cells and GFP^+^ infected cells and determined the mean fluorescence intensity (MFI) of the MHC class II cell surface expression. We did not detect any significant difference in MHC class II expression levels between uninfected and *M. tuberculosis*-infected cells three weeks post aerosol infection with virulent mycobacteria (Figure 7).

### Lung αE-DC contain the highest frequency of IL-12p40-producing cells in response to *M. tuberculosis* infection

**Figure 8:**
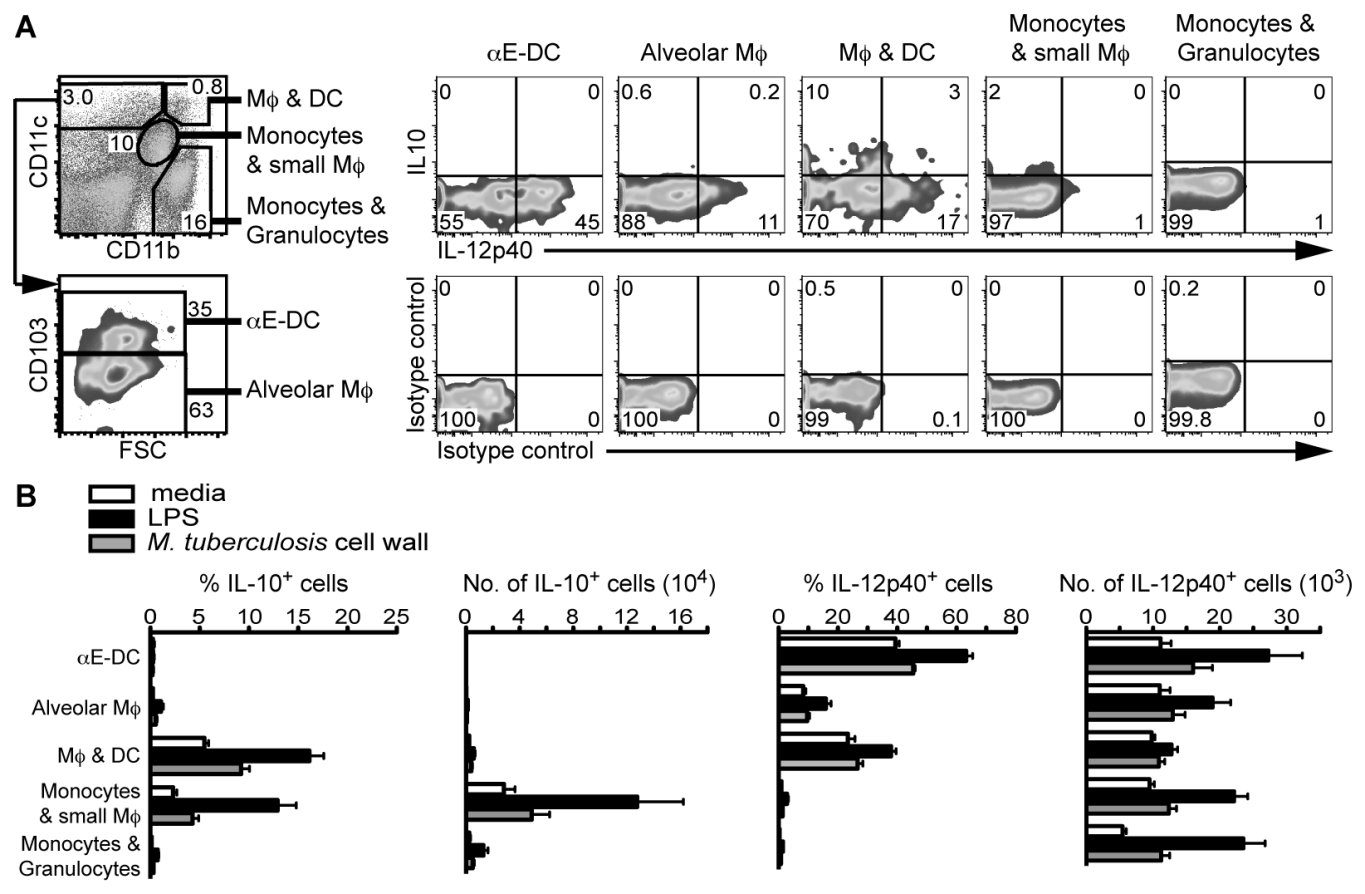
Lung αE-DC produce IL-12p40 during pulmonary TB. C57BL/6 mice were infected with a low dose of *M. tuberculosis* via the respiratory route. Three p.i., total lung cells were kept in media, or stimulated with LPS or *M. tuberculosis* cell wall extract and analyzed for intracellular IL-10 and IL-12p40. (A) The cell surface was stained for CD45.2 and CD19 (data not shown), and CD11b, CD11c and CD103. IL-10 and IL-12p40 production was compared between αE-DC, alveolar Mϕ, the subset containing both activated Mϕ and DC, and monocytes and granulocytes. The plots show *M. tuberculosis* cell wall extract-stimulated infected lung cells from one representative experiment. (B) The bar graphs show the percentage and absolute number of IL-10 and IL-12p40-producing myeloid cells three to six weeks p.i. IL-10 and IL-12p40 production by αE-DC was determined in two separate experiments with 2-3 mice per group in each experiment. The results have been pooled from all replicate experiments. The graphs display mean ± SEM.

Since protective immunity against *M. tuberculosis* is dependent on IL-12p40 production [20], we wanted to determine the main source of IL-12p40 in the *M. tuberculosis*-infected lungs three to six weeks p.i. (Figure 8). The single cell suspensions prepared from total lung tissue were kept in media alone, or stimulated with LPS or *M. tuberculosis* cell wall extract. The main myeloid cell subsets were then identified as described in Figure 2 and we determined the frequency and the absolute number of IL-10 and IL-12p40-producing cells (Figure 8).

αE-DC, alveolar Mϕ and CD11b^+^CD11c^-^ monocytes/granulocytes were essentially negative for IL-10. In contrast, CD11b^+^CD11c^+^ Mϕ/DC and CD11b ^intermediate^CD11c^intermediate^ monocytes/small Mϕ did contain detectable IL-10-producing cells, especially after LPS stimulation (Figure 8B).

Strikingly, αE-DC contained a significantly higher percentage of IL-12p40-producing cells compared to all other myeloid cell subsets, irrespective if the cells were kept in media alone, stimulated with LPS or the *M. tuberculosis* cell wall extract (p<0.001 by one-way ANOVA with Bonferroni posttest) (Figure 8B). *M. tuberculosis* cell wall extract stimulation did not significantly increase the frequency of IL-12p40 producing αE-DC, alveolar Mϕ or Mϕ/DC. By comparison, LPS stimulation lead to an increased percentage of IL-12p40^+^ cells in all three subsets. After αE-DC, CD11b^+^CD11c^+^ Mϕ/DC contained the highest percentage of IL-12p40^+^ cells. We also found that IL-10 and IL-12p40 were mainly produced by separate CD11b^+^CD11c^+^ cell subsets (Figure 8A).

In summary, we extend our previous findings on the cytokine profile of αE-DC during pulmonary TB and show that αE-DC are potent IL-12p40-producing, but not IL-10-producing cells in the *M. tuberculosis*-infected lungs [16].

## Discussion

Non-hematopoietic cells are posed to influence the outcome of *M. tuberculosis* infection [32]. Given that stromal cells, including lung epithelial cells, shape the DC phenotype [33-35], and considering the unique functional potential of αE-DC in *M. tuberculosis*-infected lungs [16], we determined the tissue localization of lung αE-DC in infected mice to be in close proximity to the bronchial epithelial cell layer and vascular wall, similar to lung αE-DC localization in uninfected lungs (Figure 4) [15]. We also detected an increased number of αE-DC in the BAL fluid from *M. tuberculosis*-infected mice similar to what has been described in the BAL from mice exposed to cigarette smoke [36]. Our original observation was that monocytes recruited into *M. tuberculosis*-infected lungs differentiate into CD11b^+^CD11c^+^ cells 6-10 days after cell transfer [3]. The differentiated cells lacked expression of typical Mϕ cell surface markers and in the present study we have shown that this population contain monocyte-derived αE-DC (Figure 2). The late appearance of monocyte-derived αE-DC compared to other DC and Mϕ subsets was a puzzling observation, but may reflect the need for the recruited monocytes to localize in close proximity to, and interacting with, bronchial epithelial cells expressing E-cadherin on the basolateral side [37,38]. The airway epithelium can produce soluble factors that influence DC recruitment. For example, CCL2 and CCL20 production by airway epithelial cells may help specify the tissue localization of CCR2^+^ monocytes, or immature DC during inflammatory conditions [39,40]. Lung epithelial cells may also provide imprinting signals and contribute to the functional potential of DC in the inflamed lungs, including GM-CSF and thymic stromal-derived lymphopoietin [34,41]. Soluble GM-CSF can also differentiate CX _3_CR1 ^+^ c-kit^+^ bone marrow cells into CD11c^+^CD103^+^ DC in vitro [42]. Thus, the bronchial epithelium may provide imprinting signals for αE-DC differentiation in the lungs just as epithelial cells in the gut-associated lymphoid tissue support tolerogenic DC formation in the gut mucosa [43].

The role of GM-CSF in αE-DC differentiation and function is not fully delineated. While an important role for GM-CSF was described by Greter et al [44], and by Unkel et al using a murine model of influenza infection [35], Edelson and colleagues presented data arguing against an essential role for GM-CSF in lung αE-DC development, even though αE-DC unable to respond to GM-CSF did express reduced cell surface levels of CD103 [45].

Jakubzick et al has shown that CCR2^+^ inflammatory monocytes give rise to lung αE-DC during steady state conditions [46]. In figure 2 we show that monocyte-derived CD11b^-^CD11c^+^ cells appearing in *M. tuberculosis*-infected lungs 10 days after cell transfer also contain αE-DC. Similar to the infected lungs, recruited monocytes upregulate high cell surface levels of CD11c within four days in infected PLN. By day 10, the cells have given rise to a monocyte-derived CD11b^-^CD11c^+^ DC subset that express CD83 and high levels of CD86, indicative of a mature DC phenotype [3]. In the present study we show that the monocyte-derived CD11b^-^CD11c^+^ DC subset in the infected PLN contain αE-DC expressing high cell surface levels of MHC class II (Figure 3).

The exact phenotype of the DC that have been implicated in disseminating live mycobacteria to the draining PLN to initiate the adaptive T cell response has not been fully defined, but the CD11b^+^CD11c^+^ DC subset is a potential candidate [2,30,47]. As αE-DC can localize beneath the bronchial epithelial cell layer and express tight junction proteins that allow αE-DC to sample the airways for inert antigens, or infectious microorganisms [15], we used GFP-expressing *M. tuberculosis* to investigate if αE-DC are targeted by the bacterium in vivo, and therefore likely candidates for mediating bacterial spread and T cell priming. Because we used a low dose aerosol infection model, we were not able to detect cells containing mycobacteria within the first week of infection. Instead, we settled for week three p.i. to examine which myeloid cells were infected in the lungs and PLN (Figure 5). Our results clearly show that only a minor fraction of αE-DC is *M. tuberculosis*-infected at this early timepoint and these cells are therefore less likely to transport the pathogen to the PLN (Figure 6).

Still, a role for αE-DC in T cell priming after *M. tuberculosis* infection cannot be fully ruled out since this DC subset is able to take up apoptotic cells in the lungs and transfer the antigens to the PLN to prime specific T cells [48]. Mutations in transcription factors required for proper DC development and function can provide further clues to the role of these cells during mycobacterial infections. The basic leucine zipper transcription factor Batf3 is required for the development of migratory αE-DC [17]. Tussiwand et al recently described reduced serum levels of IL-12p40 in *M. tuberculosis*-infected Batf3^-/-^ mice [49]. Activation of *M. tuberculosis*-specific T cells was not addressed in the absence of Batf3, but animal survival was not negatively affected [49]. Considering the importance of the MHC class II-restricted T cell response to control *M. tuberculosis* growth [31], it seems likely that CD4^+^ T cell priming is sufficient in Batf3^-/-^ mice during TB, similar to what has been described in animal models of West Nile virus infection and experimental autoimmune encephalitis [45,50]. Following low dose *M. tuberculosis* aerosol infection, the absence of CD8^+^ T cell is less dramatic [31]. Impaired, or delayed, activation of the MHC class I-restricted *M. tuberculosis*-specific CD8^+^ T cell response cannot be ruled out in Batf3^-/-^ mice.

The GFP-expressing mycobacteria also enabled us to investigate if MHC class II cell surface expression is downregulated on infected cells compared to uninfected cells in vivo. Three weeks p.i. we did not detect any significant difference in MHC class II expression levels between infected and uninfected lung cells (Figure 7). Other investigators used a similar approach to examine if live virulent *M. tuberculosis* modulates MHC class II expression on myeloid cells in infected lungs [2,22]. Kincaid et al reported no difference in MHC class II cell surface expression levels between infected and uninfected CD11b ^medium^CD11c^low^ cells, which they referred to as recruited Mϕ, 21, 28 and 35 days p.i. The authors also showed that on day 21 p.i., infected CD11b^high^CD11c^high^ cells, referred to as myeloid DC, expressed significantly higher levels of MHC class II. However, no difference in MHC class II cell surface expression was observed on infected and uninfected CD11b^high^CD11c^high^ cells 28 and 35 days p.i [22]. When Wolf et al compared MHC class II cell surface expression on infected myeloid cell subsets in the lungs over time (14-28 days p.i.), the results showed that the expression levels were not reduced, and could even increase on infected neutrophils [2]. There is also in vivo data on MHC class II expression after BCG-GFP infection. Pecora et al infected mice with 2000-4000 CFU and by day 28 p.i. the authors observed approximately 40% reduction in MHC class II expression levels on lung CD11b^high^CD11c^negative-intermediate^ Mϕ and CD11b^high^CD11b^high^ DC [51]. We speculate that the difference in mycobacterial strains used, or in the number of bacteria seeded into the lungs, may explain the different results regarding MHC class II expression levels on myeloid cell subsets in infected lungs. We therefore conclude that our results on the MHC class II expression levels on infected and uninfected myeloid cells in the lungs are comparable to previously published results using virulent *M. tuberculosis*. In vitro experiments have demonstrated that *M. tuberculosis* can interfere with several aspects of MHC class II-mediated antigen processing (reviewed in 52). Even though infected myeloid cells in vivo do not dramatically change MHC class II cell surface expression levels within the first weeks of infection, virulent mycobacteria may still inhibit activation of MHC class II-restricted CD4^+^ T cells [2].

One drawback with our experimental approach was that we were unable to investigate MHC class II cell surface expression levels on infected cells during chronic TB. At this later stage of the disease, MHC class II levels in infected lungs have been reported to be downregulated, even though no distinction was made between uninfected and *M. tuberculosis*-infected cells [29]. Taken together, monocytes recruited into the uninfected, or *M. tuberculosis*-infected lung tissue, are able to interact with multiple non-hematopoietic cells that can influence myeloid cell differentiation. Interaction with lung epithelial cells may help explain the formation of monocyte-derived αE-DC with a unique functional potential during TB. 
